# Prevalence and risk factors associated with under-five mortality in the Solomon Islands: an investigation from the 2015 Solomon Islands demographic and health survey data

**DOI:** 10.1016/j.lanwpc.2023.100691

**Published:** 2023-01-23

**Authors:** Lydia S. Kaforau, Gizachew A. Tessema, Jonine Jancey, Hugo Bugoro, Gavin Pereira

**Affiliations:** a Department of Paediatrics and Neonatal Care, National Referral Hospital, Solomon Islands; b Curtin School of Population Health, Curtin University, Perth, Australia; c School of Public Health, The University of Adelaide, South Australia, Australia; d Faculty of Nursing, Medicine and Health Sciences, Solomon Islands National University, Solomon Islands; e Centre for Fertility and Health (CeFH), Norwegian Institute of Public Health, Oslo, Norway; f enAble Institute, Curtin University, Perth, Australia

**Keywords:** Under-five mortality, Neonatal mortality, Infant mortality, Child mortality, Risk factors

## Abstract

**Background:**

Annually, over five million children die before their fifth birthday worldwide, with 98% of these deaths occurring in low-and middle-income countries. The prevalence and risks for under-five mortality are not well-established for the Solomon Islands.

**Methods:**

We used the Solomon Islands Demographic and Health Survey 2015 data (SIDHS 2015) to estimate the prevalence and risk factors associated with under-five mortality.

**Findings:**

Neonatal, infant, child and under-five mortality prevalence were 8/1000, 17/1000, 12/1000 and 21/1000 live births, respectively. After adjustment for potential confounders, neonatal mortality was associated with no breastfeeding [aRR 34.80 (13.60, 89.03)], no postnatal check [aRR 11.36 (1.22, 106.16)], and Roman Catholic [aRR 3.99 (1.34, 11.88)] and Anglican [aRR 2.78 (0.89, 8.65); infant mortality to no breastfeeding [aRR 11.85 (6.15, 22.83)], Micronesian [aRR 5.54 (1.67, 18.35)], and higher birth order [aRR 2.00 (1.03, 3.88)]; child mortality to multiple gestation [aRR 6.15 (2.08, 18.18)], Polynesian [aRR 5.80 (2.48, 13.53)], and Micronesian [aRR 3.65 (1.46, 9.10)], cigarette and tobacco [aRR 1.77 (0.79, 3.96)] and marijuana use [aRR 1.94 (0.43, 8.73)] and rural residence [aRR 1.85 (0.88, 3.92)]; under-five mortality to no breastfeeding [aRR 8.65 (4.97, 15.05)], Polynesian [aRR 3.23 (1.09, 9.54)], Micronesian [aRR 5.60 (2.52, 12.46)], and multiple gestation [aRR 3.34 (1.26, 8.88)]. Proportions of 9% for neonatal and 8% of under-five mortality were attributable to no maternal tetanus vaccination.

**Interpretation:**

Under-five mortality in the Solomon Islands from the SIDHS 2015 data was attributable to maternal health, behavioural, and sociodemographic risk factors. We recommended future studies to confirm these associations.

**Funding:**

No funding was declared to support this study directly.

**Research in context**

**Evidence before this study**

• Globally, over five million children under five years are dying annually, with over 98% of these deaths occurring in low and middle-income countries (LMICs).
• Most studies in LMICs from outside the Pacific regions and the Solomon Islands have reported that the relative risk of under-five mortality far exceeds those in high-income countries for many risk factors.

**Added value of this study**

• The prevalence of neonatal, infant, child and under-five mortality from the SIDHS 2015 data were 8/1000, 17/1000, 12/1000, and 21/1000 live births, respectively.
• Neonatal mortality was associated with no breastfeeding, no postnatal check, and practising the Roman Catholic and Anglican faiths.
• Infant mortality was associated with no breastfeeding, Micronesian ethnicities, and higher birth order.
• Child mortality was associated with multiple gestations, Polynesian and Micronesian ethnicities.
• Under-five mortality was associated with no breastfeeding, Polynesian and Micronesian ethnicities and multiple gestations.
• Approximately 41% of child mortality was attributable to being from a rural area.

**Implications of all the available evidence**

This study uses the most recent Solomon Island and Demographic and Health Survey data to provide significant information on perinatal risk factors contributing to under-five mortality in the Solomon Islands. Our results serve as a benchmark for comparison to future studies, including future studies on the influence of the pandemic.

## Introduction

Globally, approximately five million children die before their fifth birthday every year, with 98% of these deaths occurring in low and middle-income countries (LMICs).^1^^,^^2^ Under-five mortality can also be sub-classified as neonatal mortality (death within 28 days of birth), infant mortality (death within one year of birth), and child mortality (death between 1 and 5 years).^1^, ^2^, ^3^ After 25 years of global efforts guided by the fourth Millennium Development Goals (MDG) – to reduce child mortality by two-thirds by 2015 – most LMICs have still not accomplished this target. Although the global trend for under-five mortality indicates a 59% decline during this period,^4^ this reduction was trivial in LMICs.^5^^,^^6^ For instance, in 2019, more than five million children under the age of five years in LMICs died, with neonates accounting for 47% of these deaths, indicating that under-five deaths remain a significant global public health problem.^1^^,^^6^ The contribution of adverse birth outcomes to under-five mortality is high in LMICs. Notably, 90% of low birth weight (LBW) and premature neonates die in the neonatal period due to the lack of life-saving medical equipment.^3^^,^^7^ Furthermore, survivors are prone to an elevated risk of death before the age of five due to underlying preventable risks, including malnutrition and infection, increasing the child mortality totals.^7^

Following the MDG, the Sustainable Development Goals (SDG) were launched to expand and continue the achievements of the MDGs. Through the SDGs, the United Nations and World Health Organisation aimed to reduce global neonatal mortality to 12 deaths per 1000 live births and under-five mortality to 25 deaths per 1000 live births by 2030.^8^ To achieve these goals and monitor progress it is critical to first benchmark current prevalence at a national level and identify risk factors as well as the relative contributions of these risk factors to mortality. While studies have addressed this in LMICs,^5^^,^^9^, ^10^, ^11^, ^12^, ^13^ there remains a substantial knowledge gap for the Solomon Islands.

The Solomon Islands is a small developing state in the South Pacific, a double-chained archipelago with a population of 721 455 people dispersed over 992 islands.^14^^,^^15^ Most of the population (74%) live in rural areas and rely on subsistence farming and fisheries for survival.^15^ The country's predominant ethnicity is Melanesian which makes up 95% of the population.^14^ The remaining Islanders' identify as Polynesian and Micronesian.^14^ More than 90% of the Solomon Islanders are Christians, with 33% identifying as Anglicans,19% as Roman Catholics,19% as South Seas Evangelicals,12% as Seventh-day Adventists, and 10% as United Churches members.^14^

The country is known for its political instability, fragile economy and fragmented health care system.^16^ It is geographically located in one of the most vulnerable regions of the globe, with ongoing threats of natural disasters and topographical isolation, which compounds the risks of poor pregnancy and perinatal outcomes.^16^ Social and economic instability has led to a high cost of living, widespread poverty, and poor health and social infrastructure,^17^^,^^18^ which increases predisposition to neonatal, infant, child and under-five mortality.^16^ The national health strategic plan 2016 to 2020 planned to reduce under-five mortalities to 15/1000 live births,^19^ yet progress cannot be monitored because a thorough investigation of the current prevalence of under-five mortality has not yet been undertaken. Furthermore, the pertinent risk factors that contribute to neonatal, infant, child and under-five mortality in the Solomon Islands have not been fully elucidated, partly due to the lack of high-quality research and health information systems in the region. Given the underprivileged social and health structures of the population, the prevalence of under-five mortality can be substantially high, and risk factors can be associated with poor socioeconomic status, poor access to health services, and widespread poverty. The objective of this study was to estimate prevalence, risk factors and population attributable risk for neonatal, infant, child and under-five mortality using nationally representative data from the Solomon Islands Demographic and Health Survey conducted in 2015 (SIDHS 2015).

## Methods

### Study design and data source

Our study used the latest available birth data from SIDHS 2015, a population-based cross-sectional nationally representative survey conducted between 6th April to 18th September 2015. The survey's objectives were to provide current and reliable data on reproductive, maternal and child health. The SIDHS 2015 used the demographic health survey (DHS) international standard questionnaire, which was adapted for the Solomon Islands by the Solomon Islands National Statistic Office in collaboration with the Health Ministry and the Secretariat of the Pacific Community.

### Sampling and sample size

**Fig. 1:**
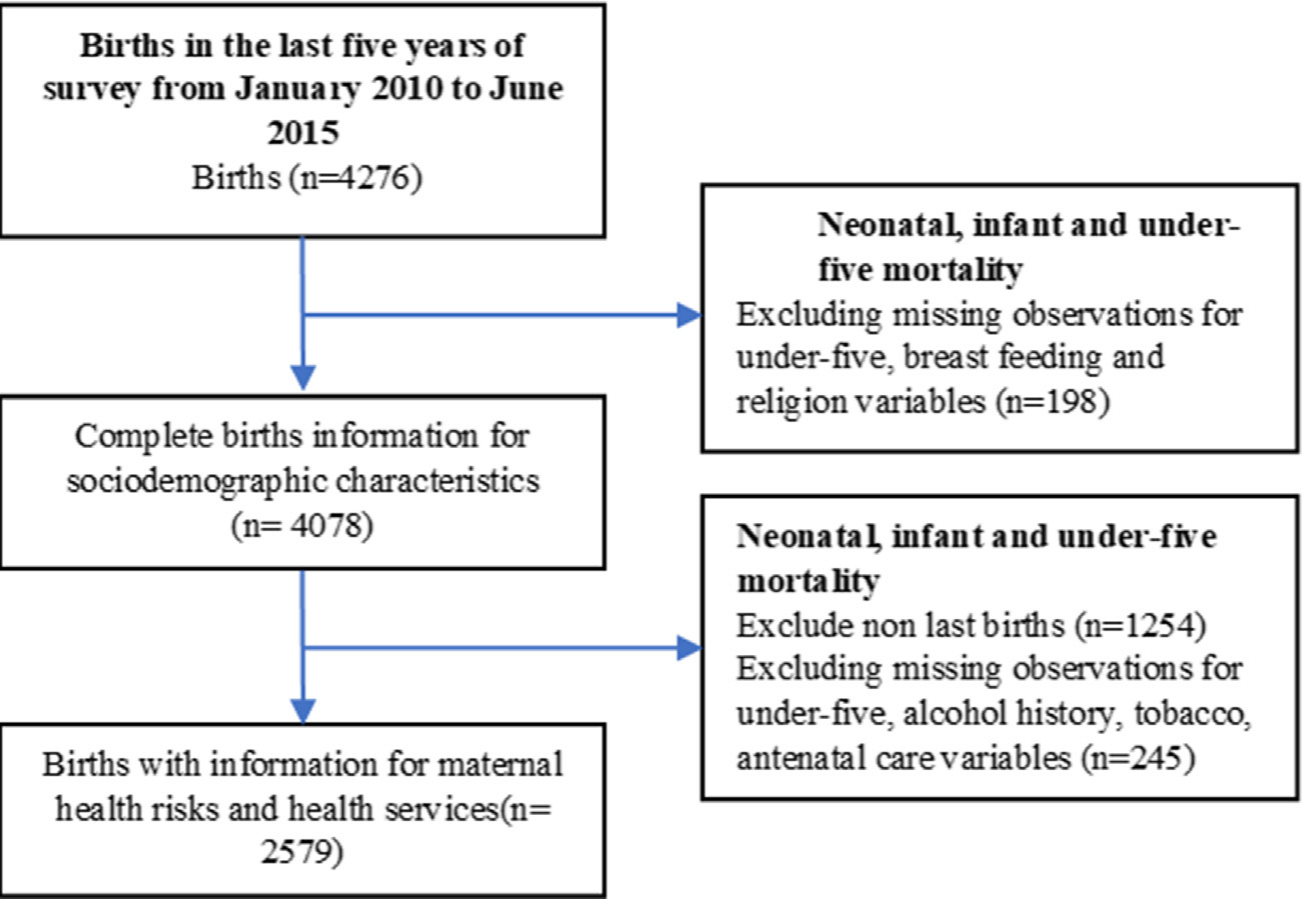
Flow chart to show showing samples included in the analysis (unweighted).

Study participants were selected from a two-stage stratified, nationally representative sample. In the first stage, enumeration areas (EAs) from the 2009 national census were selected from five provinces (Honiara; Guadalcanal; Western and Malaita Provinces) and a combination of smaller provinces (Choiseul, Santa Isabel, Central, Makira/Ulawa, Rennell/Bellona and Temotu). The sampling frame consisted of 211 EAs (primary sampling units), and EAs were selected from each province using systematic random sampling with probability proportional to the estimated number of households.^14^ In the second stage, within each EA, 24 households were selected by the survey team supervisor using systematic random sampling and participants were randomly invited from these selected households.^14^ A total of 6266 women aged 15–49 years from 5042 households were interviewed. All women with children aged 0–5 years were included. Of these women, 4272 women had births in the past 5 years at the time of completing the survey. We excluded missing observations for under-five mortality, breastfeeding and religion (n = 198), which resulted in a final unweighted sample of 4078 women. We also defined a population for a separate analysis of women with antenatal information, which required exclusion of missing observations for under-five, alcohol history, tobacco and antenatal care (n = 245) and resulted in a final unweighted sample of 2579 women (Fig. 1).

### Outcome and exposure variables

The outcome variables of interest for this study were neonatal (death before one month), infant (death before one year), child (death between 1 year and five years) and under-five mortality (all deaths before five years).^11^ We included 25 exposure variables with measures relating to sociodemographic: (marital status, religion, ethnicity, household wealth, place of residence, maternal age, maternal education, household member), maternal health risks and health services: (birth order, antenatal care, malaria in pregnancy, postnatal check, place of delivery, mode of delivery, maternal tetanus vaccination and birth attendant), child-related risk factors: (sex of child, infant weighing less than 2.5 kg or low birth weight (LBW) infant, plurality, breastfeeding), and behavioural factors: (history of tobacco and cigarette, alcohol, kava, marijuana and betel nut use). Variables selection was based on those relevant to the local context and hypothesized in the literature to be associated with neonatal, infant, child and under-five mortality. The assessment on the impact of substance use (marijuana, betel nut, kava and alcohol) assumed that women who have used any of these in the last 30 days or one year prior to the interview had an increased risk of exposure during the last pregnancy. Postnatal check variable refers to postnatal check within two months of birth (Table 1).

**Table 1:** Sociodemographic, behavioural, health and reproductive characteristics for women of the 2015 SIDHS.

Risk factors	Frequency (%)
*Sociodemographic and behavioural characteristics*	*n = 4334*

**Marital status**	
In union	3940 (90.96)
Not in union	392 (9.04)
**Religion**^a^	
Anglican	1315 (30.36)
Roman Catholic	965 (22.28)
Protestant & Pentecostal churches	1699 (39.20)
Minor religion	352 (8.15)
**Ethnicity**	
Melanesian	4183 (96.55)
Polynesian	88 (2.03)
Micronesian	57 (1.31)
Missing∗	5 (0.11)
**Household wealth**	
Poor (Quintiles 1–2)	1956 (45.16)
Middle (Quintile 3)	844 (19.49)
High (Quintiles 4–5)	1531 (35.35)
**Place of residence**	
Urban	761 (17.57)
Rural	3571 (82.43)
**Maternal age (years)**	
<20	1070 (6.04)
20–34	2242 (70.53)
35–49	1020 (23.43)
**Maternal education**	
Primary and lower	2498 (57.67)
Secondary and higher	1829 (42.23)
Missing∗	5 (0.10)
**Household member**	
1–5	1633 (37.70)
>5	2699 (62.30)
**Birth order**	
1st children	675 (15.58)
2nd −4th children	2499 (57.69)
5th children and above	1158 (26.74)
**Sex of child**	
Male	2248 (51.89)
Female	2084 (48.11)
**Plurality**	
Singleton	4256 (98.25)
Multiple gestation	746 (1.75)
**Breastfeeding**^b^	
Yes	4097 (94.58)
No	235 (5.42)
**Tobacco/cigarette use history**^c^	
Yes	732 (16.90)
No	3592 (82.91)
Missing∗	8 (0.19)
**Alcohol use history**^c^	
Yes	252 (5.81)
No	4047 (93.41)
Missing∗	34 (0.78)
**Kava use history**^c^	
Yes	113 (2.64)
No	4156 (97.19)
Missing∗	7 (0.16)
**Marijuana use history**^c^	
Yes	114 (2.67)
No	4156 (97.19)
Missing∗	6 (0.14)
**Betel nut use history**^c^	
Yes	3630 (84.89)
No	643 (15.04)
Missing∗	3 (0.07)
*Maternal health risks and health services*	*(n = 2706)*

**Antenatal care**	
Yes	2582 (95.39)
No	125 (4.61)
**Malaria in pregnancy**	
Yes	201 (9.08)
No	2008 (90.92)
Missing∗	496 (18.34)
**Postnatal check**^d^	
Yes	1716 (63.68)
No	979 (36.32)
Missing∗	10 (0.36)
**Place of delivery**	
Health facility	2338 (87.03)
Non health facility	348 (12.97)
Missing∗	17 (0.66)
**Birth weight (kilograms)**	
<2.5 kg	233 (8.82)
≥2.5 kg	2058 (76.09)
Missing∗	414 (15.30)
**Birth attendant**^e^	
High skilled health professional	2084 (78.19)
Low or no skilled attendant	581 (21.81)
Missing∗	40 (1.47)
**Mode of delivery**	
C-section	182 (7.83)
Normal vaginal delivery	2147 (92.17)
Missing∗	376 (13.89)
**Maternal tetanus vaccination**^f^	
Yes	2366 (87.48)
No	331 (12.24)
Missing∗	8 (0.28)

Study population consists of all births of sociodemographic, behavioural characteristics (weighted n = 4334) and last births of health and reproductive characteristics (weighted n = 2706) born between 2010 and 2015 of the Solomon Islands demographic health survey.

All frequencies were rounded to the nearest whole number and proportions were rounded to 2 decimal places.

∗Asterix (∗) means missing observations.

SSEC; South Seas Evangelical Church, SDA; Seventh Day Adventist.

a Minor religion refers to the combination of all minor Christian and non-Christian denominations and religions. Protestant and Pentecostal churches represent South Seas Evangelical, Seventh Day Adventist and United churches.

b Breastfeeding variable indicated if women had ever breastfed their babies.

c Tobacco and cigarette, alcohol use, kava, marijuana and betel nut use history indicated that the women had ever tried or used any of these substances in the last 30 days or 1 year of the interview.

d Postnatal check variable means child has postnatal check within 2 months of birth.

e High skilled health professionals represented, obstetricians, doctors, nurses and midwives, and low skilled represented nurse aids, and traditional birth attendants.

f Maternal tetanus vaccination during pregnancy.

### Statistical analysis

First, we summarised the distribution of background characteristics of the study population. Next, we conducted unadjusted analyses to estimate associations between these characteristics and neonatal, infant, child and under-five mortality. We also applied post-estimation tests, and variables with *p-*values of less than 0.2 were included in the multivariate analysis. We applied a generalised linear model with log link functions for the final selected covariates to estimate relative risks at a 95% confidence intervals for neonatal, infant, child and under-five mortality. This was done for complete birth information (sociodemographic characteristics) and last births (maternal health risks and health services) in eight separate models. We also assessed for multicollinearity using regression and variance inflation factors prior to the final analysis. Probability weighting was also applied to account for the method of sample selection and non-response rate during the survey. We also calculated population attributable fractions (PAF) to estimate the proportion of mortality attributable to the risk factors.^20^ All analyses were undertaken with Stata v17.^21^

### Ethical approval

Informed consent was obtained from the participants involved in the SIDHS 2015. Ethics approval was also obtained from the Solomon Islands Health Research and Ethics Board of the Ministry of Health and Medical Services (HRE039/19). Reciprocal ethics approval was granted from 10.13039/501100001797Curtin University Human Research Ethics Committee (HREC) HRE2020-0530) for the data to be accessed and analysed.

### Role of funding source

No funding source was involved in the study design; collection, analysis or interpretation of the data; and in writing of the manuscript or in the decision to submit the manuscript for publication.

## Results

### Characteristics of study population

We included two weighted populations of women who gave birth in the last five years (n = 4334) and women whose last birth or those with antenatal information (n = 2706) from the SIDHS 2015 data (Fig. 1). The mean age of the women was 29.8 years (SD = 6.49), and 29% were of the ages below 21 and over 34 years, 82% (n = 357) were from rural areas, and 45% (n = 1956) were from poor households (Table 1).

### Neonate, infant, child and under-five mortality prevalence

Using weighted sample, the five-year (2010–2015) prevalence estimated for neonatal mortality, infant, child and under-five mortality was 8/1000 live births, 17/1000 live births, 12/1000 live births and 21/1000 live births, respectively.

### Factors associated with neonatal mortality (death before 1 month)

After adjustment, the risks for neonatal mortality from the strongest to the least associations were as follows. Neonates who were not breastfed had 35 times higher risk [aRR 34.80 (13.60, 89.03)] of neonatal mortality than those breastfed. Neonates with no postnatal check within two months of birth were 11 times higher risk [aRR 11.36 (1.22, 106.16)] of neonatal mortality than those who had a postnatal check. Neonates of women practicing the Roman Catholic and Anglican faith were almost four times [aRR 3.99 (1.34, 11.88)] and three times higher risks [aRR 2.78 (0.89, 8.65)] respectively for neonatal mortality than those from other denominations or religions. Neonates with birth weight less than 2500 grams were almost three times higher risk [aRR 2.82 (0.61, 12.99)] of neonatal mortality than those with normal weight. Neonates of women with maternal age greater than 35 years were 1.54 times higher risk [aRR 1.54 (0.64, 3.71)] of neonatal mortality than those with age lower than 35 years. Neonates of women in a household with more than five members were 1.42 times less risk [aRR 0.58 (0.28, 1.21)] of neonatal mortality than those from a household of less than five members (Supplementary file 1).

### Factors associated with infant mortality (death before 1 year)

After adjustment, the risks for infant mortality from the strongest to the least associations were as follows. Infants not breastfed were 12 times higher risk [aRR 11.85 (6.15, 22.83)] of infant mortality than those breastfed. Infants of the Micronesian ethnicity were six times higher risk [aRR 5.54 (1.67, 18.35)] of infant mortality than their counterpart ethnicity. Women with multiple gestation were two times higher risk [aRR 2.41 (0.77, 7.59)] of infant mortality than single births. Infants with the fifth or more birth order position were two times higher risk [aRR 2.00 (1.03, 3.88)] of infant mortality than those with a birth order of less than five. Infants of women practising the Roman Catholic and Anglican faith were 1.74 times higher risk [aRR 1.74 (0.87, 3.48)] and 1.71 times higher risk [aRR 1.71 (0.85, 3.45)] respectively for infant mortality than those from another sects or religion. Infants with no postnatal check within two months of birth were 1.58 times higher risk [aRR 1.58 (0.52, 4.95)] of infant mortality than those with a postnatal check (Supplementary file 2).

### Factors associated with child mortality (death between 1 and 5 years)

After adjustments, the risk for child mortality from the strongest to the least associations was as follows. Women with multiple gestation infants were six times higher risk [aRR 6.15 (2.08, 18.18)] of infant mortality than single births. Children of women of Polynesian and Micronesian ethnicities were six times [aRR 5.80 (2.48, 13.53)] and 4 times [aRR 3.65 (1.46, 9.10)] respectively higher risk of child mortality than their counterpart ethnicity. Children not breastfed were two times higher risk [aRR 2.04 (0.75, 5.57)] of child mortality than those breastfed. Children of women with a history of marijuana use were 1.94 times higher risk [aRR 1.94 (0.43, 8.73)] of child mortality than non-users. Children of women from rural residences had 1.85 times higher risk [aRR 1.85 (0.88, 3.92)] of child mortality than those from urban residences. Children of women with a history of tobacco and cigarette use had 1.77 times higher risk [aRR 1.77 (0.79, 3.96)] of child mortality than non-users (Supplementary file 3).

### Factors associated with under-five mortality (all deaths under-five)

After adjustment, the risk for under-five mortality from the strongest to the least associations was as follows. Children not breastfed during the first six months of birth were eight times higher risk [aRR 8.65 (4.97, 15.05)] of under-five mortality than those breastfed. Children of women of Micronesian and Polynesian ethnicities were six times [aRR 5.60 (2.52, 12.46)] and three times [aRR 3.23 (1.09, 9.54)] higher risk of under-five mortality than their counterpart ethnicity, respectively. Children of women with multiple gestation had three times higher risk [aRR 3.34 (1.26, 8.88)] of under-five mortality than those with singleton births. Children of women practising the Roman Catholic faith were 1.62 times higher risk [aRR 1.62 (0.90, 2.94)] of under-five mortality compared to non-Roman Catholics. Children of women from a household with more than five members had a 1.45 less risk [aRR 0.55 (0.33, 0.93)] of under-five mortality than those from households of less than five members (Supplementary file 4).

### Population attributable fraction for neonatal, infant, child and under-five mortality

The proportion of neonatal mortality attributable to no postnatal check within two months of birth was 79%, no breastfeeding within the first six months of birth was 65%, LBW infant was 14%, no maternal tetanus vaccination during pregnancy was 9%. The proportion of infant mortality attributable to no exclusive breastfeeding was 37%, no postnatal check was 17%, history of tobacco and cigarette use was 7%, history of marijuana use was 2%, and multiple births was 2%. The proportion of child mortality attributable to being a rural resident was 41%, history of tobacco and cigarette use was 12%, multiple gestation was 8%, no exclusive breastfeeding was 5%, and history of marijuana use was 2%. The proportion of under-five mortality attributable to not exclusively breastfeeding for the first six months was 29%, no maternal tetanus vaccination during pregnancy was 8%, history of tobacco or cigarette use were 8%, and multiple gestation were 4% (Supplementary file 5).

## Discussion

Our study estimated under-five mortality which included neonatal, infant and child mortality and the risk associated in the Solomon Islands from the SIDHS 2015 data. Risk factors identified were maternal health risk factors, including: no breastfeeding, no postnatal check, LBW infant, multiple gestation, higher birth order, maternal age greater than 35 years. Behavioural risk factors, including: a history of tobacco, cigarette and marijuana use, and sociodemographic risk factors, including women of the Roman Catholic and Anglican faith, Polynesian and Micronesian ethnicity. Although undetected in the adjusted model. Our population attributable fraction estimation revealed that neonatal and under-five mortality were attributable to no maternal tetanus vaccination. While households with more than five members had a relatively lower risk of under-five mortality than smaller households.

We found that under-five and neonatal mortality in the Solomon Islands were 21/1000 and 8/1000 live births, respectively, which surpasses the SDG targets (25/1000 live births for under-five and 12/1000 live births for neonatal mortality). However, these findings will need to be confirmed through further analysis due to the potential for recall bias.^8^ Compared to the previous 2006 SIDHS, there was a slight decline in under-five mortality (28–21/1000 live births), infant mortality (19–17/1000 live births) and neonatal mortality (9–8/1000 live births).^14^ In light of other literature from national census data, the under-five mortality trajectory in the Solomon Islands showed a decline from 38 to 21/1000 live births between 1990 and 2015.^15^^,^^22^^,^^23^ Our estimated under-five mortality is lower than Sub Saharan countries of Africa^24^^,^^25^ and PNG,^26^ however it was still higher than some countries of the Polynesia region,^27^ which signified that under-five mortality remains a public health problem for the Solomon Islands.

The lack of breastfeeding is a health risk which associated with the four mortality groups. Our findings were supported by studies in Nigeria, Ethiopia and Indonesia that non breastfed babies were at an elevated risk of under-five mortality due to sepsis and hypoglycaemia.^12^^,^^28^, ^29^, ^30^ A recent hospital-based study in the Solomon Islands showed that early initiation of breastfeeding can also be disrupted by separation of sick newborns from their mothers due to poor rooming-in facilities which increased the risk of neonatal mortality.^31^ In addition, mortality relating to breastfeeding beyond infancy in the Solomon Islands may be related to early weaning, as 21% of the infants who were weaned before the recommended six months were found to have an elevated risk of malnutrition, infection and death before the age of five years.^14^^,^^32^ Recent studies showed that 20% of infants (aged 6–23 months) and 40% of children (aged 2–5 years) in the Solomon Islands are subjected to malnutrition and death from recurrent diarrhoeal diseases.^32^^,^^33^ Therefore, no breastfeeding, late breastfeeding initiation, and early weaning are likely risk factors for neonatal and under-five mortality. Other health risks were related to maternal health and services. No postnatal check within two months of birth was strongly associated with neonatal and infant mortality and supported by studies in Indonesia, Burkina Faso and Cambodia, as early postnatal checks allow the early detection and treatment of life threatening complications in infants.^34^^,^^35^ Higher birth order (fifth and more) was also a risk for death during the infancy period, which was also observed by an Indian study, which proposed mothers to be less cautious with high birth order, compared to low birth order infants.^36^ Multiple gestation was also found to be a risk factor for infant, and under-five mortality, confirmed by studies in Ghana and Ethiopia, and deaths were due to congenital anomalies, poor birth outcomes and challenges with feeding.^37^, ^38^, ^39^, ^40^, ^41^

Behavioural risk factors such as a history of tobacco and cigarette use was associated with child mortality, which was also confirmed by studies from Southeast Asia.^42^^,^^43^ It is widely known that nicotine has a role in vasoconstriction of the placenta and hypoperfusion resulting in fetal growth restriction, *placenta abruption* and the risk of early infant deaths.^42^, ^43^, ^44^ Besides, studies on passive smoking also showed that children of smoking parents have an increased susceptibility to respiratory infections that may be responsible for child mortality.^42^^,^^43^ Given the high prevalence of smoking among women in the Solomon Islands (57%), tobacco smoke is a notable behavioural risk factor for child mortality in this population.^45^ The history of marijuana use was also a behavioural risk factor associated with child mortality. Although studies on marijuana in LMICs are limited, studies in the United States have showed that child mortality due to marijuana use can be due to intrauterine, breastmilk and passive aerosolized exposure.^46^, ^47^, ^48^, ^49^ *Cannabinoid,* the main active ingredient of marijuana, has been posited to interfere with fetal-placental circulation in pregnancy, provoking intrauterine growth restriction or inflicting harm to young children via breastmilk and aerosol exposure.^46^, ^47^, ^48^, ^49^

Our study showed that sociodemographic factors, including ethnicity, religion, rural residences and maternal age greater than 35 years were associated with under-five mortality. The Polynesian and Micronesian ethnicity of a social minority (3% of the study population) showed elevated risks of under-five mortality, specifically during infancy and childhood. Although there is a dearth of literature locally, studies on ethnic minorities, including women of the Pacific Islands living in the United States, showed that ethnic minority is a risk factor for under-five mortality and other adverse birth outcomes attributed to social disparities and cultural marginalization.^50^, ^51^, ^52^, ^53^, ^54^ Women practising the Roman Catholic or Anglican faiths had a higher risk of under-five mortality, especially during the neonatal and infancy period, than their counterpart sects and religion (Protestant and Pentecostal churches and minor religions). Although there is a paucity of literature on under-five mortality specific to these religions, studies in Mozambique have also hypothesized that affiliation to a particular religion, for instance, those connected to the health sector, could be in a better position for child survival.^55^ Children of women from rural residences were associated with child mortality, which was supported by a study in Burkina Faso due to poor access to health facilities.^56^ Advanced maternal age was also a risk for neonatal mortality, supported by a study in several African countries, as with advanced age, women are vulnerable to obstetric and lifestyle diseases, which can complicate pregnancy.^57^, ^58^, ^59^, ^60^ Given the high incidence of non-communicable diseases in the Solomon Islands,^14^ advanced maternal age is a notable risk factor for neonatal mortality.

Interestingly, our results showed that larger households (>5 members) had a lower risk of neonatal and under-five mortality, which contradicts most studies in LMICs. For instance, studies in African countries showed that large families, possibly of a high parity, are a risk factor for under-five and infant mortality.^28^^,^^61^^,^^62^ Although, living arrangements may be comparable to the Solomon Islands, larger households in the African context may result in competition for food and therefore inadequate nutrition, which has been linked to poor pregnancy outcomes and deaths.^28^ Alternatively, larger households in the Solomon Islands may be advantageous, as they may enable social support and security, which is tied to the wantok system, a socio-economic and political network commonly practiced in the Solomon Islands and Melanesia.^63^ Therefore living in an extended family household may be an indication of an advantage for support and protection, thereby reducing the risk of neonatal and under-five mortality.^14^ We suggest further studies evaluate the role of the wantok system on maternal and child health.

The PAFs were estimated to range from 7% to 79% for neonatal, infant, child, or under-five mortality. High PAFs were estimated for no post-natal check, no breastfeeding, rural residence, LBW infant, history of tobacco and cigarette use, multiple gestations, which were also confirmed by our adjusted analysis. Although maternal tetanus vaccination did not show an association in the adjusted analysis, proportions of 9% and 8% of neonatal and under-five mortality were attributable to no tetanus vaccinations, respectively. Although there is a dearth of studies on neonatal and under-five tetanus infection in the Solomon Islands, studies in the LMICs of Nepal and PNG showed that a lack of maternal tetanus vaccination was associated with neonatal and under-five mortality*.*^11^^,^^64^

### Strengths and limitations

The SIDHS 2015 study used a representative sample, which was the study's strength. However, there were also limitations as shared by all demographic and health surveys. Recall bias common to all retrospective studies can be a significant limitation to estimate the under-five mortality and the risk factors. Misreporting the date of birth and age at death due to shifting age or heaping to years before 2010 can distort the indices deviating the estimation to the true value. Recall bias can affect variables such as maternal tetanus vaccination, malaria infection, and breastfeeding as mothers may not give correct information. Social desirability bias can also potentially affect our study due to barriers such as low literacy and the lack of information on births. Women may provide incorrect information about their age and underreporting or denial of illegal substance use, including marijuana or *kwaso* (home-brewed distilled alcohol) and betel nut, beer or kava. Also, as most Solomon Islanders live in extended families, the number of people living in a household fluctuates and could either be under or overestimated. Another limitation is that the SIDHS 2015 data is not current. However, population-level characteristics do not tend to vary over relatively short periods, and consequently, the Demographic and Health Surveys are conducted at 5–10 year intervals. Nonetheless, our results will serve as a benchmark for comparison in future studies, including future studies on the influence of the pandemic.

### Conclusion

The prevalence of neonatal, infant, child and under-five mortality based on the SIDHS 2015 data were 8/1000, 17/1000, 12/1000 and 21/1000 live births, respectively. Furthermore, under-five mortality and its subgroups from the SIDHS 2015 data were associated with maternal health, behavioural and sociodemographic risk factors. Given the high chance that under-five mortality is underestimated, further prospective studies with a nationally represented sample and robust methodology are required to confirm these findings. Future studies can now assess the direct and indirect impacts of the SARS-CoV-2 pandemic on perinatal and child health using these results as a benchmark.

## Contributors

LSK, GT, JJ and GP conceived and conceptualised study design. LSK conducted data cleaning, merging and analysis under GT and GP's guidance. LSK did the final selection of study variable under the guidance of GT and GP. LSK wrote first draft and reviewed by GT, JJ, GP and HB. All authors have approved the final paper.

## Patient and public involvement

No patient was directly involved in the study as data were taken from the SIDHS.

## Data sharing statement

The Solomon Island Government, through the Solomon Island National Statistic Office (SINSO), kept all the data that supports the findings of this study. Data can be obtained upon formal request in writing to SINSO's Government statistician.

## Declaration of interests

The authors declared no potential conflicts of interest.
