# Supplementary material for: Prevalence and risk factors associated with under-five mortality in the Solomon Islands: an investigation from the 2015 Solomon Islands demographic and health survey data
Source: Lancet Reg Health West Pac. 2023 Jan 23;33:100691. doi: 10.1016/j.lanwpc.2023.100691 (PMC10166993; doi:10.1016/j.lanwpc.2023.100691)
Supplement: Supplementary material [file mmc1.pdf]

Supplementary files

Table of Contents

Supplementary file 1. Unadjusted and adjusted analysis of sociodemographic, maternal health and behavioural risks for neonatal mortality of the 2015 SIDHS. 2

Supplementary file 2. Unadjusted and adjusted analysis of sociodemographic, maternal health and behavioural risks for infant mortality of the 2015 SIDHS. 4

Supplementary file 3. Unadjusted and adjusted analysis of sociodemographic, maternal health and behavioural risks for child mortality of 2015 SIDHS. 6

Supplementary file 4. Unadjusted and adjusted analysis of sociodemographic, maternal health and behavioural risks for under-five mortality of 2015 SIDHS. 8

Supplementary file 5. Population attributable fractions of sociodemographic, maternal health and behavioural risks for neonatal, infant, child and under-five mortality in the Solomon Islands 10

Supplementary file 1. Unadjusted and adjusted analysis of sociodemographic, maternal health and behavioural risks for neonatal mortality of the 2015 SIDHS.

COVARIATE AND GROUP	NEONATAL MORTALITY			
Sociodemographic, behavioural, health characteristics, weighted population (n = 4334)				
	Number (%)		OR 95% CI	
	No (%)	Yes (%)	CRR	ARR
**Marital status**				
In union	3910(90.94)	30(93.10)	**Reference**	
Not in union	390(9.06)	2(6.90)	0.74 (0.16, 3.47)	
**Religion**[1]				
Anglican	1302(30.27)	14(42.93)	3.12 (0.99, 9.89)	2.78 (0.89, 8.65)
Roman Catholic	954(22.19)	11(33.97)	3.36 (1.17, 9.70)	3.99 (1.34, 11.88)
Protestant & Pentecostal churches	1693(39.36)	6 (17.75)	**Reference**	**Reference**
Minor religion	351(8.17)	2(5.35)	1.45 (0.29, 7.36)	1.62 (0.34, 7.66)
**Ethnicity**				
Melanesian	4152(96.57)	30(94.69)	**Reference**	
Polynesian	87(2.02)	1(2.98)	1.51 (0.16, 13.93)	
Micronesian	56(1.31)	1(2.33)	1.81 (0.22, 15.09)	
**Wealth quintile**				
Poor (Quintiles 1-2)	1939(45.11)	17(52.63)	1.00 (0.41, 2.48)	0.85 (0.34, 2.16)
Middle (Quintile 3)	842(19.59)	2(6.37)	0.28 (0.06, 1.32)	0.22 (0.05, 0.94)
High (Quintiles 4-5)	1518(35.31)	13(41.01)	**Reference**	**Reference**
**Place of residence**				
Urban	755(17.55)	7(20.24)	**Reference**	
Rural	3545(82.45)	26(79.76)	0.84 (0.38, 1.87)	
**Maternal age (years)**				
≤20	1064(24.75)	6(17.71)	0.57 (0.07, 4.41)	0.37 (0.04, 3.18)
21-34	2227(51.80)	15(46.76)	**Reference**	**Reference**
35-49	1008(23.45)	11(35.52)	1.73 (0.82, 3.64)	1.54 (0.64, 3.71)
**Maternal education**				
Primary & lower	2481(57.70)	17(53.59)	0.84 (0.40, 1.80)	
Secondary & above	1814(42.20)	15(46.41)	**Reference**	
**Household member**				
1-5	1617(37.61)	16(49.41)	**Reference**	**Reference**
> 5	2683(62.39)	16(50.59)	0.61 (0.38, 0.97)	0.58 (0.28, 1.21)
**Birth order**				
1st children	670(15.57)	5(16.56)	1.32 (0.48, 3.61)	
2nd - 4th children	2484(57.77)	15(46.52)	**Reference**	
5th children and above	1146(26.66)	12(36.92)	1.71 (0.78, 3.75)	
**Sex of child**				
Male	2233(51.92)	15(47.45)	**Reference**	
Female	2067(48.08)	17(52.55)	1.20 (0.50,2.85)	
**Plurality**				
Single	4227(98.26)	31(97.52)		
Multiple gestation	75(1.74)	1(2.48)	1.43 (0.35, 5.78)	
**Breastfeeding**				
Yes	4086(95.04)	11(34.16)	**Reference**	**Reference**
No	213(4.96)	21(65.84)	33.68 (13.92, 81.51)	34.80 (13.60, 89.03)
**Tobacco/cigarette history**				
Yes	728 (16.94)	4(11.05)	0.61 (0.18, 2.02)	
No	3563(82.87)	29(88.95)	**Reference**	
**Alcohol use history**				
Yes	247(5.76)	4(12.73)	2.35 (0.69, 7.98)	0.58 (0.28, 1.21)
No	4019(93.46)	28(87.27)	**Reference**	**Reference**
**Kava use history**				
Yes	8(1.89)	1(2.56)	1.36 (0.19, 9.98)	
No	4210(97.93)	31(97.44)	**Reference**	
**Marijuana use history**				
Yes	92(2.10)	1(2.56)	1.20 (0.16, 8.92)	
No	4200(97.70)	31(97.44)	**Reference**	
**Betel nut use history**				
Yes	3619(85.20)	76(90.55)	1.27 (0.40, 4.01)	
No	626(14.2)	8(9.45)	**Reference**	
Health and reproductive characteristics, weighted population (n = 2706)				
**Antenatal care**				
Yes	2323(87.11)	37 (91.64)	2.99 (0.39, 23.05)	
No	344(12.89)	3(8.36)	**Reference**	

**Malaria infection**				
Yes	200(7.45)	1(3.29)	0.48 (0.06, 3.74)	
No	1992(74.29)	16(68.28)	**Reference**	
**Postnatal check**				
Yes	1714(63.91)	2(9.91)	**Reference**	**Reference**
No	959(35.77)	19(84.45)	14.95 (3.12, 71.65**)**	11.36 (1.22, 106.16)
**Place of delivery**				
Health facility	1714(63.91)	2(9.91)	**Reference**	
Non health facility	959(35.77)	19(84.45)	0.46 (0.07, 2.89)	
**Birth attendant**				
High skilled	2063(76.92)	21(90.34)	**Reference**	
Low or no skilled	579(21.60)	2(9.66)	2.61 (0.65, 10.44)	
**Birth weight (kilograms)**				
<2.5 kilograms	230(8.59)	3(11.86)	2.86 (0.64, 12.78**)**	2.82 (0.61, 12.99)
≥2.5 kilograms	2050(76.42)	8(36.65)	**Reference**	**Reference**
**Mode of delivery**				
C-section	178(6.64)	4(18.30)	2.86 (0.81, 10.06**)**	1.06 (0.13, 8.83)
Normal vaginal delivery	2130(79.41)	17(75.32)	**Reference**	**Reference**
**Tetanus vaccination**				
Yes	2348(87.57)	18(76.76)	**Reference**	**Reference**
No	326(12.15)	5(23.24)	2.15 (0.77, 6.05)	1.79 (0.19, 16.53)

Footnotes: Population consists of all births (weighted n=4334) and last births (weighted n=2706) born between 2010 and 2015 of the Solomon Islands demographic health survey.
Adjusted analyses were made for all covariates with p-values less than 0.2
Frequencies (n) were rounded to the nearest whole number
Abbreviations: CRR; crude relative risk, ARR; adjusted relative risk.

Supplementary file 2. Unadjusted and adjusted analysis of sociodemographic, maternal health and behavioural risks for infant mortality of the 2015 SIDHS.

**COVARIATE AND GROUP**	**INFANT MORTALITY**			
Sociodemographic, behavioural, health characteristics, weighted population (n = 4334)				
	**Number (%)**		**RR 95% CI**	
	**No (%)**	**Yes (%)**	**CRR**	**ARR**
**Marital status**				
In union	3881(90.92)	62(91.35)	**Reference**	
Not in union	388(9.08)	6(8.65)	0.95 (0.39, 2.33)	
**Religion**				
Anglican	1290(30.22)	25(37.74)	1.76 (0.80, 3.83)	1.71 (0.85, 3.45)
Roman Catholic	948(22.20)	19(28.40)	1.80 (0.88, 3.66)	1.74 (0.87, 3.48)
Protestant & Pentecostal churches			**Reference**	**Reference**
Minor religion¹	349(8.17)	4(6.09)	1.06 (0.32, 3.44)	1.19 (0.41, 3.43)
**Ethnicity**				
Melanesian	4125(96.63)	62(91.75)	**Reference**	**Reference**
Polynesian	86(2.02)	2(2.55)	1.32 (0.24, 7.29)	1.32 (0.17, 10.28)
Micronesian	53(1.24)	4(5.71)	4.57 (1.75, 11.94)	5.54 (1.67, 18.35)
**Household wealth**				
Poor (Quintiles 1-2)	1921(45.00)	37(55.30)	1.13 (0.60, 2.14)	1.11 (0.54, 2.30)
Middle (Quintile 3)	841(19.70)	4(6.59)	0.31 (0.10, 0.96)	0.27 (0.09, 0.82)
High (Quintiles 4-5)	1507(35.30)	26(38.11)	**Reference**	**Reference**
**Place of residence**				
Urban	752(17.61)	10(15.30)	**Reference**	
Rural	3517(82.39)	57(84.70)	1.18 (0.64, 2.18)	
**Maternal age (years)**				
≤20	257(6.02)	3(3.68)	0.59 (0.17, 2.09)	
21-34	3006(70.42)	50(73.66)	**Reference**	
35-49	1006(23.57)	15(22.66)	0.92 (0.53, 1.60)	
**Maternal education**				
Primary & lower	2460(57.63)	40(60.06)	1.10 (0.62, 1.94)	0.49 (0.27, 0.87)
Secondary & above	1804(42.26)	27(39.94)	**Reference**	**Reference**
**Household member**				
1-5	1601(37.51)	32(48.27)	**Reference**	
> 5	2667(62.49)	35(51.73)	0.65 (0.40, 1.06)	
**Birth order**				
1st children	665(15.59)	9(14.13)	1.09 (0.53, 2.25)	1.48 (0.69, 3.19)
2nd - 4th children	2469(57.85)	32(47.94)	**Reference**	**Reference**
5th children and above	1334(26.56)	25(37.93)	1.71 (0.93, 3.15)	2.00 (1.03, 3.88)
**Sex of child**				
Male	2217(51.95)	34(50.80)	**Reference**	
Female	2051(48.05)	33(49.20)	1.05 (0.54, 2.03)	
**Plurality**				
Single	4196(98.31)	64(94.80)	**Reference**	**Reference**
Multiple gestation	72(1.69)	3(5.20)	3.08 (1.13, 8.40)	2.41 (0.77, 7.59)
**Breastfeeding**				
Yes	4059(95.10)	42(62.14)	**Reference**	**Reference**
No	209(4.90)	25(37.86)	10.64 (5.65, 20.04)	11.85 (6.15, 22.83)
**Tobacco/cigarette use history**				
Yes	718(16.82)	16(23.43)	1.50 (0.74, 3.01)	1.42 (0.66, 3.08)
No	3542(82.99)	51(76.57)	**Reference**	**Reference**
**Alcohol use history**				
Yes	245(5.74)	7(9.72)	1.73 (0.74, 4.08)	1.22 (0.43, 3.45)
No	3988(93.46)	61(90.28)	**Reference**	**Reference**
**Kava use history**				
Yes	81(1.89)	2(2.35)	1.24 (0.30, 5.16)	
No	4178(97.93)	66(97.65)	**Reference**	
**Marijuana use history**				
Yes	90(2.11)	3(4.06)	1.93 (0.60, 6.20)	1.60 (0.31, 8.25)
No	4169(97.72)	64(95.94)	**Reference**	**Reference**
**Betel nut use history**				
Yes	3638(85.26)	59(88.19)	1.28 (0.50, 3.30)	
No	626(14.66)	8(11.81)	**Reference**	
Health and reproductive characteristics, weighted population (n = 2706)				
**Antenatal care**				

Yes	2545(95.43)	37(92.56)	**Reference**	
No	122(4.57)	3(7.44)	1.66 (0.52, 5.29)	
**Malaria in pregnancy**				
Yes	199(7.47)	2(4.06)	0.53 (0.13, 2.23)	
No	1977(74.20)	31(76.93)	**Reference**	
**Postnatal check**				
Yes	1702(63.84)	16(39.68)	**Reference**	**Reference**
No	956(35.86)	23(56.33)	2.49 (1.14, 5.43)	1.58 (0.52, 4.95)
**Place of delivery**				
Health facility	2303(86.37)	37(92.68)	**Reference**	
Non health facility	346(12.96)	3(7.32)	0.53 (0.15, 1.83)	
**Birth attendant**				
High skilled	2049(76.83)	37(91.76)	**Reference**	
Low or no skilled	578(21.68)	3(8.24)	0.32 (0.10, 1.04)	
**Birth weight (kilograms)**				
<2.5 kilograms	228(8.55)	5(13.18)	2.31 (0.78, 6.89)	1.18 (0.18, 7.97)
≥2.5 kilograms	2037(76.47)	20(50.37)	**Reference**	**Reference**
**Mode of delivery**				
C-section	177(6.65)	5(12.40)	1.82 (0.59, 5.63)	0.30 (0.02, 3.74)
Normal vaginal delivery	2116(79.37)	32(80.28	**Reference**	**Reference**
**Maternal tetanus vaccination**				
Yes	2330(87.58)	36(81.17)	**Reference**	**Reference**
No	323(12.13)	8(18.83)	1.59 (0.63, 4.05)	1.45 (0.31, 6.75)

Footnotes: Population consists of all births (weighted n=4334) and last births (weighted n=2706) born between 2010 and 2015 of the Solomon Islands demographic health survey.
Adjusted analyses were made for all covariates with p-values less than 0.2
Frequencies (n) were rounded to the nearest whole number
Abbreviations: CRR; crude relative risk, ARR; adjusted relative risk.

Supplementary file 3. Unadjusted and adjusted analysis of sociodemographic, maternal health and behavioural risks for child mortality of 2015 SIDHS.

COVARIATE AND GROUP	CHILD MORTALITY			
Sociodemographic, behavioural, health characteristics, weighted population (n = 4334)				
	Number (%)		RR 95% CI	
	No (%)	Yes (%)	CRR	ARR
**Marital status**				
In union	3,865(90.95)	45(88.56)	**Reference**	
Not in union	385(9.05)	6(11.44)	1.29 (0.52, 3.22)	
**Religion**				
Anglican	1,286(30.26)	15(30.12)	1.01 (0.46, 2.24)	
Roman Catholic	942(22.17)	14(26.53)	1.21 (0.61, 2.43)	
Protestant & Pentecostal churches	1673(39.36)	20(38.71)	**Reference**	
Minor religion¹	349(8.21)	2(4.64)	0.58 (0.12, 2.76)	
**Ethnicity**				
Melanesian	4,109 (96.69)	44(85.94)	**Reference**	**Reference**
Polynesian	84(1.97)	3(6.34)	3.53 (1.65, 7.56)	3.65 (1.46, 9.10)
Micronesian	52(1.23)	4(7.72)	6.64 (2.86, 15.39)	5.80 (2.48, 13.53)
**Household wealth**				
Poor (Quintiles 1-2)	1913(45.02)	28(54.72)	1.25 (0.62, 2.49)	
Middle (Quintile 3)	837(19.69)	6(10.93)	0.57 (0.18, 1.82)	
High (Quintiles 4-5)	1500(35.29)	18(34.35)	**Reference**	
**Place of residence**				
Urban	748(17.61)	6(10.88)	**Reference**	
Rural	3501(82.39)	46(89.12)	1.74 (0.79, 3.86)	1.85 (0.88, 3.92)
**Maternal age (years)**				
≤20	255(6.00)	3(6.48)	0.92 (0.31, 2.71)	0.97 (0.33, 2.86)
21-34	2993(70.42)	42(83.11)	**Reference**	**Reference**
35-49	1002(23.58)	5(10.41)	0.38 (0.13, 1.06)	0.43 (0.16, 1.16)
**Maternal education**				
Primary & lower	2453(57.71)	29(57.09)	0.97 (0.51, 1.87)	
Secondary & above	1973(42.18)	22(42.91)	**Reference**	
**Household member**				
1-5	1592(37.45)	25(49.21)	**Reference**	**Reference**
> 5	2658(62.55)	26(50.79)	0.62 (0.34, 1.13)	0.62 (0.33, 1.17)
**Birth order**				
1st children	664(15.62)	6(11.31)	0.73 (0.25, 2.10)	
2nd - 4th children	2456(57.80)	29(57.39)	**Reference**	
5th children and above	1130(26.58)	16(31.30)	1.18 (0.53, 2.64)	
**Sex of child**				
Male	2205(51.88)	58(57.84)	Reference	
Female	2045(48.12)	22(42.16)	0.79 (0.39, 1.59)	
**Plurality**				
Single	4179(98.34)	47(91.74)	**Reference**	**Reference**
Multiple gestation	71(1.66)	4(8.26)	5.08 (1.71, 15.06)	6.15 (2.08, 18.18)
**Breastfeeding**				
Yes	4042(95.10)	46(91.00)	**Reference**	**Reference**
No	208(4.90)	5(9.00)	1.90 (0.69, 5.22)	2.04 (0.75, 5.57)
**Tobacco/cigarette use history**				
Yes	713(16.79)	16(31.49)	2.24 (1.11, 4.54)	1.77 (0.79, 3.96)
No	3528(83.02)	35(68.51)	**Reference**	**Reference**
**Alcohol use history**				
Yes	244(5.73)	4(7.58)	1.33 (0.49, 3.62)	
No	3972(93.47)	47(92.42)	**Reference**	
**Kava use history**				
Yes	80(1.89)	1(2.15)	1.14 (0.24, 5.46)	
No	4162(97.93)	50(97.85)	**Reference**	
**Marijuana use history**				
Yes	89(2.10)	3(5.66)	2.74 (0.75, 10.05)	1.94 (0.43, 8.73)
No	4154(97.74)	48(94.34)	**Reference**	**Reference**
**Betel nut use history**				
Yes	3621(85.20)	47(91.98)	1.97 (0.64, 6.10)	1.44 (0.46, 4.57)
No	626(14.72)	4(8.02)	**Reference**	**Reference**
Health and reproductive characteristics, weighted population (n = 2706)				
**Antenatal care**				
Yes	2540(95.42)	21(100.0)	**Reference**	

No	122(4.58)	0(0.00)		
**Malaria in pregnancy**				
Yes	198(7.44)	1(5.83)	0.66 (0.13, 3.34)	0.52 (0.17, 1.66)
No	1976(74.22)	19(89.00)	**Reference**	**Reference**
**Postnatal check**				
Yes	1699(63.83)	17(79.69)	**Reference**	
No	955(35.87)	4(18.86)	0.42 (0.14, 1.24)	
**Place of delivery**				
Health Facility	2298(86.34)	19(92.99)		
Non health facility	355(12.98)	1(7.01)	0.50 (0.10, 2.61)	
**Birth attendant**				
High skilled	228(8.56)	3(12.22)	**Reference**	
Low or no skilled	2035(76.45)	16(74.57)	0.19 (0.03, 1.50)	
**Birth weight (kilograms)**				
<2.5 kilograms	2044(76.79)	20(94.83)	1.46 (0.31, 6.79)	
≥2.5 kilograms	578(21.72)	1(5.17)	**Reference**	
**Mode of delivery**				
C-section	175(6.58)	5(11.36)	1.67 (0.33, 8.38)	
Normal vaginal delivery	2114(79.42)	17(81.63)	**Reference**	
**Maternal tetanus vaccination**				
Yes	2330(87.53)	36(81.1)	**Reference**	
No	324(12.19)	8(18.83)	1.23 (0.29, 5.17)	

Footnotes: Population comprised of all births (weighted n=4334) and last births (weighted n=2706) born between 2010 and 2015 of the Solomon Islands demographic health survey.
Adjusted analyses were made for all covariates with p-values less than 0.2
Frequencies (n) were rounded to the nearest whole number
Abbreviations: CRR; crude relative risk, ARR; adjusted relative risk.

Supplementary file 4. Unadjusted and adjusted analysis of sociodemographic, maternal health and behavioural risks for under-five mortality of 2015 SIDHS.

COVARIATE AND GROUP	UNDER-FIVE MORTALITY			
Sociodemographic, behavioural, health characteristics, weighted population (n = 4334)				
	Number (%)		RR 95% CI	
	No (%)	Yes (%)	CRR	ARR
**Marital status**				
In union	3865(90.98)	75(89.57)	**Reference**	
Not in union	383(9.02)	9(10.43)	1.17 (0.55, 2.47)	
**Religion**				
Anglican	1286(30.28)	29(34.78)	1.44 (0.76, 2.72)	1.34 (0.74, 2.42)
Roman Catholic	941(22.14)	24(29.16)	1.64 (0.92, 2.95)	1.62 (0.90, 2.94)
Protestant & Pentecostal churches			**Reference**	**Reference**
Minor religion¹	349(8.21)	4(4.87)	0.75 (0.25, 2.27)	0.83 (0.31, 2.25)
**Ethnicity**				
Melanesian	4107(96.69)	75(89.41)	**Reference**	**Reference**
Polynesian	84(1.97)	4(5.00)	2.66 (1.26, 5.61)	3.23 (1.09, 9.54)
Micronesian	52(1.23)	5(5.59)	4.59 (2.46, 8.58)	5.60 (2.52, 12.46)
**Household wealth**				
Poor (Quintiles 1-2)	1912(45.00)	45(53.46)	1.12 (0.64, 1.96)	1.14 (0.61, 2.13)
Middle (Quintile 3)	837(19.69)	8(9.09	0.44 (0.18, 1.11**)**	0.41 (0.17, 1.00)
High (Quintiles 4-5)	1500(35.31)	31(37.45	**Reference**	**Reference**
**Place of residence**				
Urban	748(17.62)	13(15.21)	**Reference**	
Rural	3500(82.38)	71(84.79)	1.19 (0.70, 2.03)	
**Maternal age (years)**				
≤20	1053(24.80)	17(20.00)	0.81 (0.32, 2.02)	
21-34	2192(51.61)	50(59.21)	**Reference**	
35-49	1002(23.59)	17(20.79)	0.84 (0.50, 1.41)	
**Maternal education**				
Primary & lower	2451(57.70)	47(56.10)	0.94 (0.58, 1.52)	
Secondary & above	1792(42.20)	37(43.90)	**Reference**	
**Household member**				
1-5	1592(37.46)	42(49.71)	**Reference**	**Reference**
> 5	2657(62.54)	42(50.29)	**0.**61 (0.38, 0.97)	0.55 (0.33, 0.93)
**Birth order**				
1st children	664(15.62)	11(13.23)	1.08 (0.53, 2.18)	
2nd - 4th children	2455(57.78)	44(52.74)	**Reference**	
5th children and above	1130(26.59)	29(34.03)	1.50 (0.71, 3.18)	
**Sex of child**				
Male	2203(51.86)	45(53.37)	**Reference**	
Female	2045(48.14)	39(46.63)	0.94 (0.53, 1.68)	
**Plurality**				
Single	4177(98.34)	79(94.03)	**Reference**	**Reference**
Multiple gestation	71(1.66)	5(5.97)	3.57 (1.41, 9.03)	3.34 (1.26, 8.88)
**Breastfeeding**				
Yes	4040(95.10)	57(68.45)	**Reference**	**Reference**
No	208(4.90)	26(31.55)	8.05 (4.57, 14.17)	8.65 (4.97, 15.05)
**Tobacco/cigarette use history**				
Yes	712(16.75)	20(24.22)	1.56 (0.88, 2.77)	1.48 (0.81, 2.72)
No	3528(83.05)	64(75.78)	**Reference**	**Reference**
**Alcohol use history**				
Yes	244(5.73)	8(9.49)	1.69 (0.79, 3.60	1.24 (0.52, 2.97)
No	3971(93.47)	76(90.51)	**Reference**	**Reference**
**Kava use history**				
Yes	80(1.89)	2(2.29)	1.21 (0.35, 4.16)	
No	4160(97.93)	82(97.71)	**Reference**	
**Marijuana use history**				
Yes	89(2.10)	4(4.42)	2.11 (0.71, 6.26)	1.35 (0.34, 5.35)
No	4152(97.74)	80(95.58)	**Reference**	**Reference**
**Betel nut use history**				
Yes	3619(85.20)	76(90.55)	1.64 (0.64, 4.21)	1.03 (0.39, 2.70)
No	626(14.72)	8(9.45)	**Reference**	**Reference**
Health and reproductive characteristics, weighted population (n = 2706)				
**Antenatal care**				
Yes	2540(95.42)	42(93.32)	**Reference**	

No	122(4.58)	3(6.68)	1.48 (0.47, 4.70)	
**Malaria infection**				
Yes	198(7.44)	3(5.99)	0.78 (0.27, 2.30)	
No	1974(74.20)	34(76.92)	**Reference**	
**Postnatal check**				
Yes	1697(63.81)	19(42.44)	**Reference**	**Reference**
No	595(35.89)	24(53.98)	2.23 (1.09, 4.56)	1.38 (0.58, 3.26)
**Place of delivery**				
Health facility	2297(86.33)	42(93.42)	**Referenc**e	
Non health facility	346(12.99)	3(6.58)	0.47(0.14, 1.62)	
**Birth attendant**				
High skilled	2042(76.77)	41(92.60)	**Reference**	
Low or no skilled	578(21.73)	3(7.40)	0.29 (0.09, 0.93)	
**Birth weight (kilograms)**				
<2.5 kilograms	228(8.56)	5(11.84)	1.89 (0.66, 5.45)	1.07 (0.16, 7.03)
≥2.5 kilograms	2033(76.43)	25(55.40)	**Reference**	**Reference**
**Mode of delivery**				
C-section	175(6.58)	7(16.33)	2.50 (0.97, 6.42)	1.13 (0.22, 5.76)
Normal vaginal delivery	2112(79.41)	34(77.09)	**Reference**	**Reference**
**Maternal tetanus vaccination**				
Yes	2330(87.58)	36(81.17)	**Reference**	**Reference**
No	323(12.13)	8(18.83)	1.65 (0.69, 3.93)	1.67 (0.45, 6.14**)**

Footnotes: Population comprised of all births (weighted n=4334) and last births (weighted n=2706) born between 2010 and 2015 of the Solomon Islands demographic health survey.
Adjusted analyses were made for all covariates with p-values less than 0.2
Frequencies (n) were rounded to the nearest whole number
Abbreviations: CRR; crude relative risk, ARR; adjusted relative risk.

Supplementary file 5. Population attributable fractions of sociodemographic, maternal health and behavioural risks for neonatal, infant, child and under-five mortality in the Solomon Islands

Groups at risk	Proportion exposed to risks (%)	Adjusted relative risk	Population attributable fraction (%)
**Neonatal mortality**			
No postnatal check	36.32	11.36	79
No breastfeeding	5.42	34.8	65
LBW infant	8.82	2.82	14
No tetanus vaccination	12.24	1.79	9
**Infant mortality**			
No breastfeeding	5.42	11.85	37
Postnatal check	36.32	1.58	17
Tobacco/cigarette use history	16.9	1.42	7
Marijuana use history	2.67	1.6	2
Multiple births	1.75	2.41	2
**Child mortality**			
Rural Residence	82.43	1.85	41
Tobacco/cigarette use history	16.9	1.77	12
Multiple gestation	1.75	6.15	8
No breastfeeding	5.42	2.04	5
Marijuana use history	2.67	1.94	2
**Under-five mortality**			
No breastfeeding	5.42	8.65	29
No tetanus vaccination	12.24	1.67	8
Tobacco/cigarette use history	16.9	1.48	8
Multiple births	1.75	3.34	4
